# Network Pharmacology and Pharmacological Evaluation Reveals the Mechanism of the *Sanguisorba Officinalis* in Suppressing Hepatocellular Carcinoma

**DOI:** 10.3389/fphar.2021.618522

**Published:** 2021-03-04

**Authors:** Nan Jiang, Hong Li, Yueshan Sun, Jing Zeng, Fei Yang, Fahsai Kantawong, Jianming Wu

**Affiliations:** ^1^ Department of Medical Technology, Faculty of Associated Medical Sciences, Chiang Mai University, Chiang Mai, Thailand; ^2^ School of Pharmacy, Southwest Medical University, Luzhou, China; ^3^ International Education School, Southwest Medical University, Luzhou, China; ^4^ Education Ministry Key Laboratory of Medical Electrophysiology, Sichuan Key Medical Laboratory of New Drug Discovery and Drugability Evaluation, Luzhou Key Laboratory of Activity Screening and Drugability Evaluation for Chinese Materia Medica, Southwest Medical University, Luzhou, China

**Keywords:** PI3K/Akt signal pathway, cell proliferation, network pharmacology, hepatocellular carcinoma, *Sanguisorba officinalis* L, EGFR/MAPK signaling

## Abstract

**Background:** *Sanguisorba Officinalis* L. (SO) is a well-known traditional Chinese medicine (TCM), commonly applied to treat complex diseases, such as anticancer, antibacterial, antiviral, anti-inflammatory, anti-oxidant and hemostatic effects. Especially, it has been reported to exert anti-tumor effect in various human cancers. However, its effect and pharmacological mechanism on hepatocellular carcinoma (HCC) remains unclear.

**Methods:** In this study, network pharmacology approach was applied to characterize the underlying mechanism of SO on HCC. Active compounds and potential targets of SO, as well as related genes of HCC were obtained from the public databases, the potential targets and signaling pathways were determined by protein-protein interaction (PPI), gene ontology (GO) and pathway enrichment analyses. And the compound-target and target-pathway networks were constructed. Subsequently, *in vitro* experiments were also performed to further verify the anticancer effects of SO on HCC.

**Results:** By using the comprehensive network pharmacology analysis, 41 ingredients in SO were collected from the corresponding databases, 12 active ingredients screened according to their oral bioavailability and drug-likeness index, and 258 potential targets related to HCC were predicted. Through enrichment analysis, SO was found to show its excellent therapeutic effects on HCC through several pathways, mainly related to proliferation and survival via the EGFR, PI3K/AKT, NFκB and MAPK signaling pathways. Additionally, *in vitro*, SO was found to inhibit cell proliferation, induce apoptosis and down-regulate cell migration and invasion in various HCC cells. Moreover, western blot analysis showed that SO treatment down-regulated the expression of p-EGFR, p-PI3K, p-AKT, p-NFκB and p-MAPK proteins in HepG2 cells. These results validated that SO exerted its therapeutic effects on HCC mainly by the regulation of cell proliferation and survival via the EGFR/MAPK and EGFR/PI3K/AKT/NFκB signaling pathways.

**Conclusion:** Taken together, this study, revealed the anti-HCC effects of SO and its potential underlying therapeutic mechanisms in a multi-target and multi-pathway manner.

## Introduction

Hepatocellular carcinoma (HCC) is the sixth most commonly diagnosed cancer, ranking the third leading cause of cancer-related death according to GLOBCAN in 2018, with approximately 841,000 new incidences and 782,000 deaths yearly around the world, posing a major health problem (Bray et al., 2018; EASL, 2018). Although survival rates of HCC patients have improved on benefit from modern therapeutic strategies, namely, liver transplantation, radiofrequency ablation, transcatheter arterial chemoembolization, surgical resection, and sorafenib (Liu et al., 2015; EASL, 2019), many HCC patients still face high long-term mortality, recurrence, drug resistance and serious side effects (Cheng et al., 2009; Zhu et al., 2017). Therefore, it is urgent to search for the alternative therapeutic strategies with low toxicity and less drug resistance for HCC.

Traditional Chinese medicine (TCM) with diverse chemical substances has been widely accepted as an effective strategy with less toxicity and higher efficacy for the therapy of cancer (Yuan et al., 2017; Xiang et al., 2019). Researches shown that TCM has the advantages in clinical cancer treatment, especially in suppressing tumor cells, reducing the side effects of radiotherapy and drug resistance, and improving patients’ overall survival (Liao et al., 2017; Kuo et al., 2018; Lou et al., 2018). The plant of *Sanguisorba Officinalis* L. (SO), also known as Diyu in Chinese, a widely used herb medicine in east Asia, has been prescribed clinically for more than 2000 years in China (Zhao et al., 2017). According to preliminary reports, SO possessed a variety of pharmacological effects, such as anticancer (Cai et al., 2012), antibacterial (Chen et al., 2015), antiviral (Kim et al., 2010), anti-inflammatory (Seo et al., 2016), anti-oxidant (Zhang et al., 2012) and hemostatic effects (Sun et al., 2012). It was reported that a hot water preparation made of a single herb (SO) has excellent antitumor effect in human oral cancer cells (Shin et al., 2012). Moreover, triterpenoids and tannins isolated from the roots of SO have been shown promising antitumor effects in several cancer researches (Liu et al., 2005; Bai et al., 2019; Li et al., 2019). Notably, it was reported recently that Ziyuglycoside II, a major active compound of SO, markedly impaired HepG2 proliferation, migration and invasion, by blocking EGFR/NF-kB signaling (Liao et al., 2020). However, the effects and mechanism of SO to treat HCC have not been fully elucidated with suitable approaches.

TCM is associated with complex chemical composition and synergistic or antagonistic interactions, making it difficult to systematically study its pharmacological mechanisms with conventional pharmacological approaches (Ma et al., 2015). Fortunately, the concept of network pharmacology, as an integrated approach, derived from systems biology and bioinformatics, was proposed to comprehensively investigate and study the underlying molecular mechanisms of Chinese herb medicine (Ma et al., 2015). Network pharmacology analysis was used to reveal the complex mechanism of TCM, via the construction and visualization of “medicine-target-disease” interaction network, (Li and Zhang, 2013). In recent years, network pharmacology emerged as a powerful tool resonates well with the holistic view of TCM. This approach has been successful applied to investigate the complex mechanism of TCM in many researches (Zhang et al., 2013; He et al., 2019; Wan et al., 2019).

**FIGURE 1:**
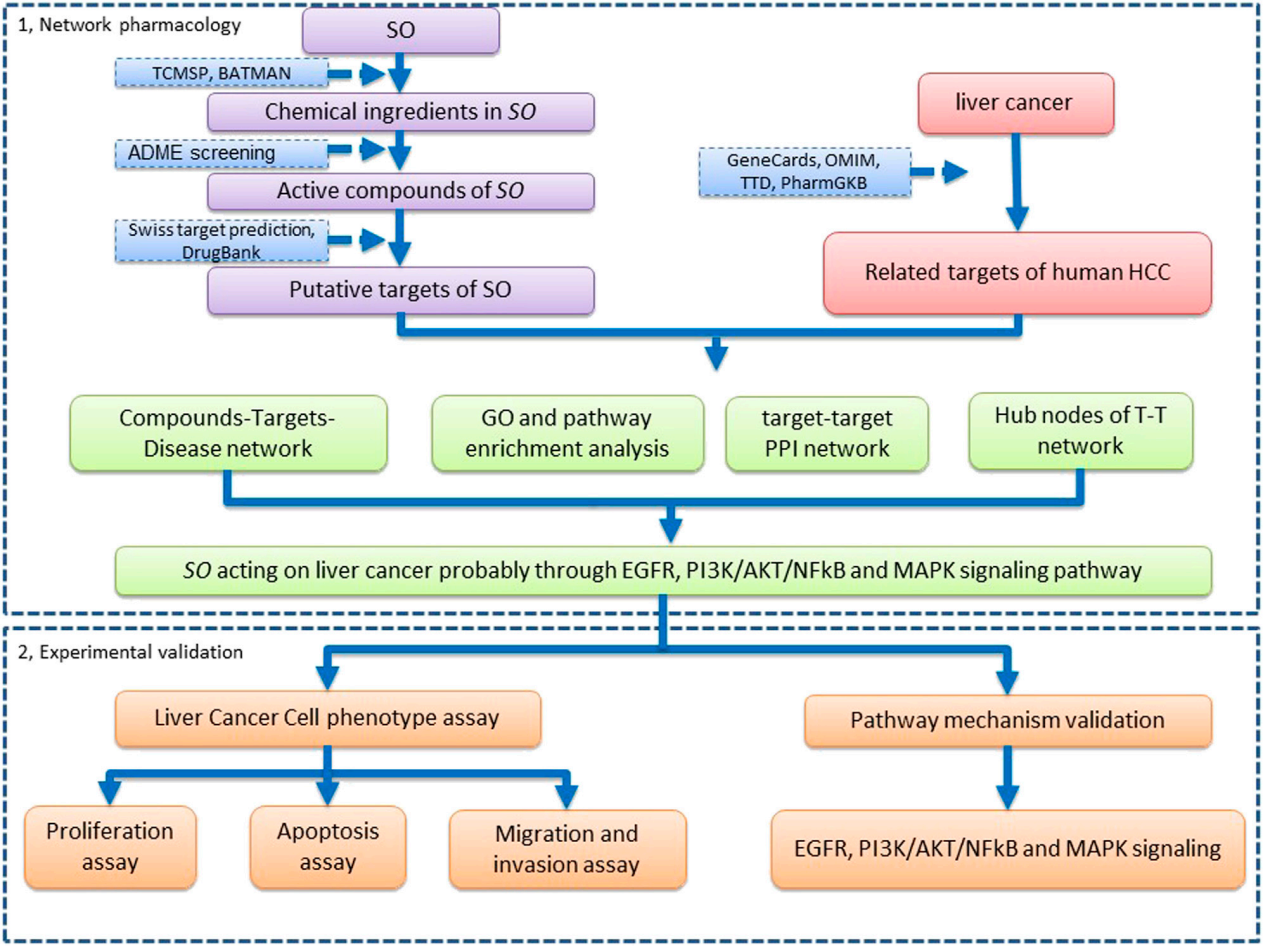
The workflow of network pharmacology analysis and validation of SO on HCC.

In the present study, we aim to construct the pharmacological network to explore the potential molecular mechanisms and pathways of SO on HCC, using multiple database and computational tools. Additionally, *in vitro* experiments were also performed to verify the underlying molecular mechanism of SO on HCC, as predicted by network pharmacology. The workflow of this study is shown in Figure 1.

## Method

### Collection of Chemical Ingredients in *Sanguisorba Officinalis* L. for Network Pharmacology Analysis

As previous reported (Ru et al., 2014), Potential candidate compounds of SO were acquired from the two following databases: 1) Traditional Chinese Medicine Systems Pharmacology Database and Analysis Platform (TCMSP, http://tcmspw.com/tcmsp.php, Version. 2.3) (Ru et al., 2014). A total of 499 registered Chinese herbal medicines and 12,144 ingredients from the Chinese Pharmacopoeia (2010 edition) were collected in the TCMSP database (Ru et al., 2014). 2) Bioinformatics Analysis Tool for Molecular mechanism of Traditional Chinese Medicine (BATMAN-TCM, http://bionet.ncpsb.org/batman-tcm, updated in 2016), including of 46,914 formulas, 8,159 medical herbs, and 25,210 components (Liu et al., 2016).

### Active Ingredients Screening Strategy

For oral traditional Chinese medicine, absorption, distribution, metabolism, and excretion (ADME) was employed to screen active components with potential therapeutic effects (Liu et al., 2013). The screening criterion we applied in this research were 1) Oral bioavailability (OB) greater than 30%, 2) Drug-likeness (DL) larger than 0.18. A total of 12 active ingredients and their corresponding 2D and 3D structures were downloaded from the PubChem database (https://pubchem.ncbi.nlm.nih.gov/) (Zhu et al., 2017).

### Prediction of Targets of the Active Ingredients in *Sanguisorba Officinalis* L.

The related protein targets of bioactive components were retrieved from TCMSP (TCMSP, http://tcmspw.com/tcmsp.php, Version. 2.3) (Ru et al., 2014) and Swiss Target Prediction network database (STP, http://www.swisstargetprediction.ch/, updated in 2019) (Gfeller et al., 2014). The UniProt database (https://www.uniprot.org/) was adopted to change protein names to their corresponding gene symbols (UniProt Consortium, 2018).

### Collection of Potential Target Genes for Hepatocellular Carcinoma

The HCC-related gene targets were collected from four databases: GeneCards (https://www.genecards.org/, version. 5.0) database (Stelzer et al., 2016). OMIM (http://www.omim.org/, updated in 2020) database (Amberger and Hamosh, 2017). Therapeutic Target Database (TTD, https://db.idrblab.net/ttd/, updated in 2020) (Li et al., 2018). PharmGKB (https:// www.pharmgkb.org/, updated in 2020) (Whirl-Carrillo et al., 2012), using keywords such as “hepatocarcinoma” and “Hepatocellular Carcinoma”.

### Construction of Protein–Protein Interaction (PPI) Network

Protein–protein interaction network was constructed by the STRING (https://string-db.org/, version. 11.0) database, using the overlap genes between SO active ingredients targets and HCC targets (Szklarczyk et al., 2019), with the species limited to “Homo sapiens”, and correlation degree greater than 0.700, as the cut-off confidence score.

### Pharmacology Network Construction

Pharmacology networks were generated as follows: 1) Active compound-Target-Disease network of SO (C-T-D network). 2) OS targets—HCC targets PPI network (T-T network). 3) Target - pathway network (T-P network). The pathway annotation of targets was obtained from the KEGG pathway enrichment analysis. All networks were visualized by Cytoscape 3.7.0 (Shannon et al., 2003).

### Network Topological Analysis

For network topological analysis, we calculated three parameters to assess topological features of nodes, namely: 1) “Degree” measures links to one node. 2) “Betweenness Centrality” is the number of a node lies on paths between other nodes. 3) “Closeness Centrality” measures the mean distance from a node to other nodes (Tang et al., 2015). The level of the three parameters shows the topological importance of the nodes in the network.

### Gene Ontology (GO) and KEGG Pathway Enrichment Analyses

Database for Annotation, Visualization and Integrated Discovery (Huang da et al., 2009) (DAVID, https://david.ncifcrf.gov/, version 6.8) was used for gene ontology (GO) and KEGG pathway enrichment analysis (Kanehisa et al., 2017).

### Reagents and Antibodies

Fetal bovine serum (FBS), Dulbecco’s modified Eagle’s medium (DMEM) were purchased from Gibco (Gibco, Thermo Fisher Scientific, Waltham, MA, United States). Penicillin and streptomycin were obtained from Beyotime (Beyotime, Sichuan, China). Paclitaxel (PTX) purchased from Sigma-Aldrich (St. Louis, MO, United States) Primary antibodies, including anti-EGFR (#4267), anti-p-EGFR (#3777), anti-PI3-kinase (#4249), anti-p-PI3K (#4228), anti-AKT (#4691), anti-p-AKT (#4060), anti-NFκB-P65 (#8242), anti-p-NFκB-P65 (#3033), anti-MAPK (#4695), anti-p-MAPK (#4370), anti-Cleaved Caspase-3 (#9664), anti-PARP (#9542) and β-actin (#4970) were purchased from Cell Signaling Technology Inc. (CST, MA, United States).

### Preparation of *Sanguisorba Officinalis* L. Ethanol Extract

Dried roots of SO were obtained from Chengdu Wukuaishi herbal medicine wholesale market (Chengdu, Sichuan, China). The original herb was authenticated by professor Can Tang of the Department of Chinese Materia Medica, Southwest Medical University, China. A voucher specimen (SWMU-YL-DY2019031501) was deposited at the specimen repository of the Department of Traditional Chinese Medicine, Southwest Medical University. Samples (200 g) of dried roots of SO were extracted with 2 L of 75% ethanol and then filtered with a 0.22 μm pore-size filter. The filtrates of ethanol extracts of SO (ESO) were evaporated to dried powder and stored in −20°C for future use.

### Phytochemical Analysis of ESO

ESO were qualitatively analyzed by ultra-high-performance liquid chromatography (Exion)—QTOF (X500R) MS system (SCIEX, MA, United States). AB Sciex ExionLC system (AB SCIEX, Foster City, CA, United States), equipped with an ExionLC Solvent Delivery System, an ExionLC AD Auto-sampler, an ExionLC AD Column oven, an ExionLC Degasser, an ExionLC AD Pump, an ExionLC PDA Detector, and an ExionLC Controller, were used for chromatographic analysis. ESO were separated on Phenomenex Kinetex C18 column (100 × 2.1 mm, 2.6 μm, 100 Å) at 40°C. The sample was eluted at a flow rate of 0.2 ml/min in a gradient elution program of A (0.1% formic acid-water (v/v)) and B (0.1% formic acid-acetonitrile(v/v)): 0–2.00 min (5% B); 2.01–18 min (5–50% B); 18.00–20.00 min (50–100% A). The injection volume was 5 μL.

### Cell Culture

Human HCC cells (HepG2, MHCC97H, SMCC7721, and BEL-7404) were chosen for the following experiments. HepG2 and SMCC7721 cells were commercially obtained from American Type Culture Collection (ATCC, Manassa, VA, United States); BEL-7404 cells were and MHCC97H cells were kindly gifted by Professor Lv Muhan from department of internal medicine, The affiliated hospital of Southwest Medical University. Cells were cultured in DMEM medium supplemented with 10% FBS, 100 U/ml penicillin-streptomycin and maintained at 37°C in a humidified incubator with 5% CO_2_.

### Cell Viability Assay

HCC cells were seeded in 96-well plates at density of 4 × 10^4^ cells/ml (100 μL/well) and incubated for 24 h. After pretreatment with different concentrations of ESO (0, 15.625, 31.25, 62.5, 125, 250, 500, and 1,000 μg/ml) for 24, and 48 h. Following the addition of 10 μL CCK8 (Dojindo, Japan) per well, cells were cultured for another 4 h at 37°C. The absorbance was determined at 450 nm using a Microplate Reader (Thermo Fisher Scientific Inc., Waltham, MA, United States).

### 5-Ethynyl-2′-deoxyuridine (EdU) Assay

The proliferation of HCC cells was determined by EdU assay. After HCC cells (4,000 cells/well) were seeded in 96-well plates and incubated for 24 h, cells were exposed to concentrations of ESO (0, 62.5, 125, and 250 μg/ml) for 24 h and incubated with 10 μM EdU (APExBIO, Houston, United States) for another 2 h. Then the cells were fixed with 3.7% formaldehyde for 15 min and cell nuclei were stained with Hoechst 33,342. Eventually, EdU-positive cells were observed and photographed under a photographed at a magnification of ×100 with an ImageXpress - Micro 4 High-Content Screening system (Molecular Devices, LLC, CA, United States). The EdU-positive cells (%) = The count of red EdU/The count of blue Hoechst 33,342 × 100.

### Wound-Healing and Transwell Invasion Assay

For wound-healing assay, HepG2 and SMCC7721 cells were incubated in 6-well plates with 100% confluence. After cells were scratched using a sterile pipette tip on the cell monolayer, medium was removed and the monolayer was washed 3 times with PBS. Then, medium containing indicated concentrations of ESO was added to each well and cell movements into the wound area were obtained after 0 and 24 h incubation with a phase-contrast inverted microscope at a magnification of ×40. The transwell invasion assay was conducted using a Corning transwell chamber system (8.0 μm, #3442, Corning, NY, United States). 1.5 × 10^5^ treated cells were seeded into the upper chamber in the presence of a Matrigel-precoated membrane (#M8370, Solarbio, Beijiang, China) containing 200 μL of serum-free MEM. Then, complete medium (500 μL) containing 10% FBS was added to the bottom chamber. Following incubation for 24 h at 37°C, the chambers were washed twice with phosphate-buffered saline, fixed with 4% paraformaldehyde, and stained with 1% crystal violet solution at room temperature. Cells were counted under a light microscope in five random fields.

### Flow Cytometry for Analysis of Cell Apoptosis

Apoptosis was analyzed using an Annexin V-FITC apoptosis detection kit (San Jose, CA, United States). HepG2 and SMCC7721 cells were seeded at a density of 1.5 × 10^5^ cells/well in 6-well plates. After 24 h treatment with ESO at indicated concentrations, the cells were analyzed using an annexin V-FITC/PI Detection Kit from BD Biosciences (San Jose, CA, United States) to detect apoptosis. The apoptotic cells were detected by FACSVerse flow cytometer (BD Biosciences, San Jose, CA, United States). Data acquisition and analysis were performed using the Flowjo software (BD Biosciences, San Jose, CA, United States).

### Western Blotting

Cells (1.5 × 10^5^ cells/well) were seeded into 6-well plates. Cell proteins were extracted after treatment of the indicated concentrations of ESO, according to procedures as described previously (Teng et al., 2020). The cells were lyzed in 1 × PIPA buffer, containing 1: 100 protease and phosphatase inhibitor cocktail on ice for 30 min. The protein concentration of the lysate was then determined by the Bradford protein assay reagent (Bio-Rad, CA, United States) according to the manufacturer’s instructions. An equal amount of the protein (30 μg per sample) was loaded onto 10% sodium dodecyl sulfate polyacrylamide gel electrophoresis (SDS-PAGE) for separation and transferred onto polyvinylidene difluoride (PVDF) membranes (Bio-Rad, United States). Then, the membranes were blocked with 5% no-fat milk for 1 h at room temperature and incubated with the primary antibodies including anti-EGFR, anti-p-EGFR, anti-PI3-kinase, anti-p-PI3-kinase, anti-AKT, anti-p-AKT, anti-NFκB (p65), anti-p-NFκB (p65), anti-MAPK, anti-p-MAPK and β-actin at 1:1,000 (Cell Signaling Technology, Danvers, MA, United States) overnight at 4°C. Then, the membranes were washed with TBST three times and further incubated with HRP-conjugated secondary antibodies for 1 h at room temperature and the protein bands were developed by an UltraSignal Hypersensitive ECL Chemiluminescent Substrate (4A Biotech Co., Ltd., Beijing, China). and detected by the ChemiDoc MP Imaging System (Bio-Rad, California, United States). The band intensity of proteins was quantified by using ImageJ software (National Institutes of Health, MD, United States), and the relative expression of protein was normalized by expression of β-actin.

### Statistical Analysis

All the data were presented as means ± standard deviation (SD). Difference between groups was analyzed by one-way univariate analysis of variance (ANOVA) by Prism 8.0 software (San Diego, CA, United States). if *p*-value < 0.05, difference was considered to be statistically significant (marked as *). Higher significance levels were established at *p*-value < 0.01 (marked as **).

## Result

### Identification and Verification of Active Ingredients of *Sanguisorba Officinalis* L.

A total of 41 ingredients of SO were retrieved by searching the TCMSP and BATMAN database (Supplementary Table S1), and 12 bioactive ingredients were preliminarily screened out using ADME parameters such as OB and DL, as shown in Table 1. And to verify those active ingredients of SO, the 12 compounds of ESO were identified by UHPLC– QTOF, shown in Supplementary Figure S1.

**TABLE 1:** Active ingredients and ADME parameters of *SO.*

Mol ID	Molecule name	Structure	OB (%)	DL
MOL000098	Quercetin	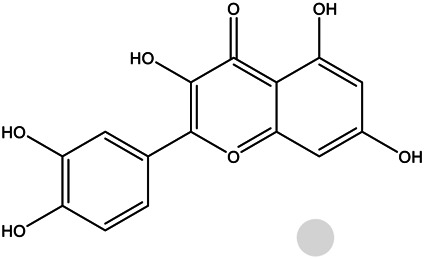	46.43	0.28
MOL000211	Mairin	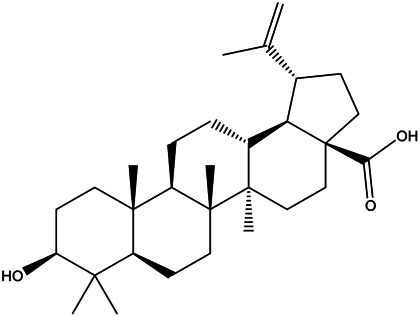	55.38	0.78
MOL000358	Beta-sitosterol	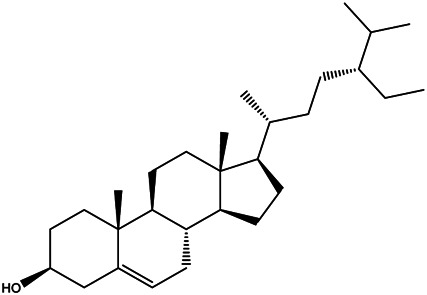	36.91	0.75
MOL000422	Kaempferol	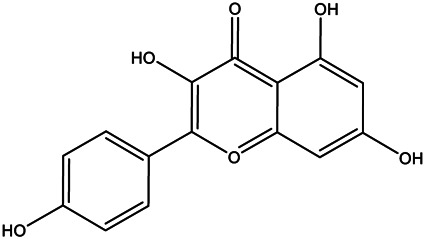	41.88	0.24
MOL005399	Alexandrin_qt	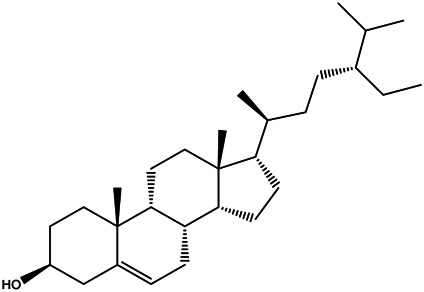	36.91	0.75
MOL005853	Methyl-2,3,6-tri-O-galloyl-β-d-glucopyranoside	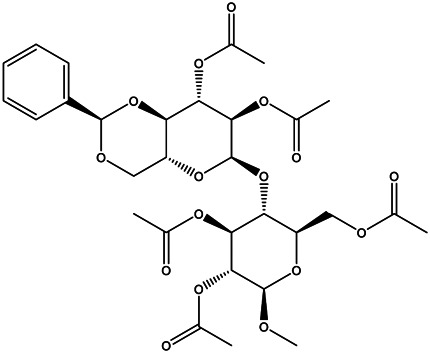	44.95	0.67
MOL005858	3,7,8-Tri-O-methylellagic acid	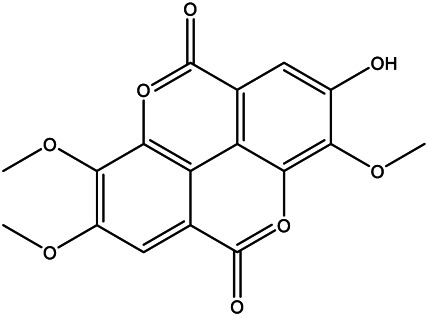	37.54	0.57
MOL005860	3-O-galloylprocyanidin B-3	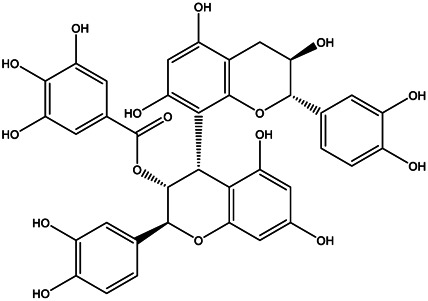	30.06	0.33
MOL005862	Methyl 4,6-di-O-galloyl-beta-d-glucopyranoside	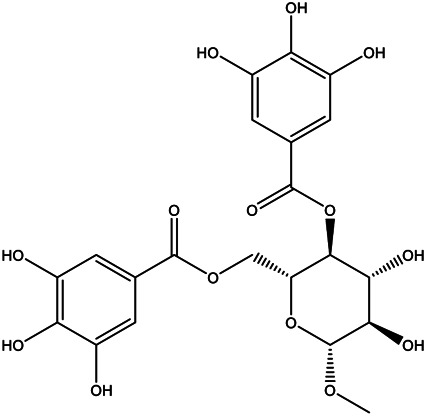	48.07	0.68
MOL005864	Methyl-6-O-galloyl-β-d-glucopyranoside	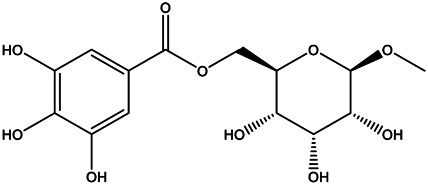	44.85	0.29
MOL005880	Sauvissimoside R1	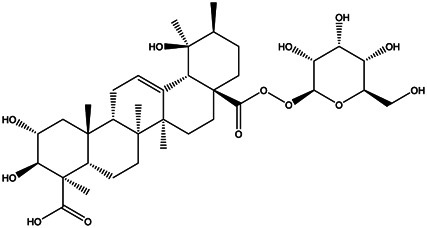	37.39	0.31
MOL005883	Gambiriin B-3	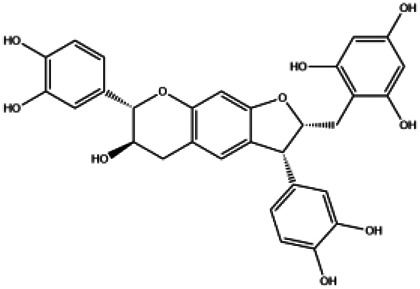	34.99	0.75

### Target Identification of Active Ingredients of *Sanguisorba Officinalis* L.

**FIGURE 2:**
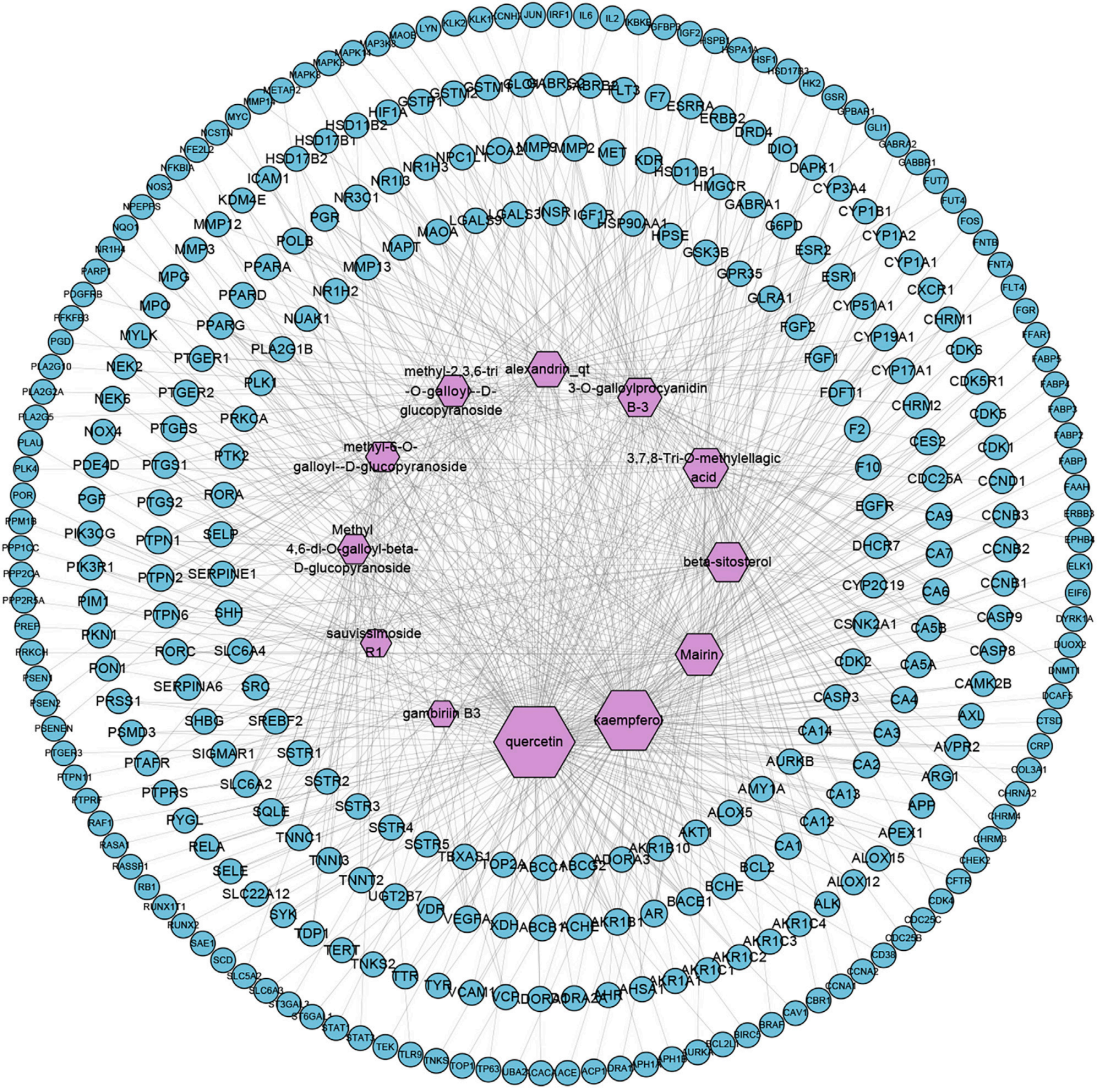
Compound-Target network of SO (C-T network). Network of 12 active ingredients of SO and 327 putative targets. The size of compound nodes is proportional to the number of degrees.

TCMSP and Swiss Target Prediction database were used to predict the targets of 12 active compounds of *SO*, and a total of 327 targets were obtained after high-possibility screening and overlaps eliminating. And we found that 201 of the 327 putative targets were common to 2 or more of these ingredients, showing that these ingredients act in similar biological processes or pathways, suggesting the synergistic effect of multiple ingredients in *SO.* The compound-target network is shown in Figure 2, and according to the number of degrees, the top four ingredients were quercetin, kaempferol, mairin, and beta-sitosterol.

### *Sanguisorba Officinalis* L. Active Compound Target-Hepatocellular Carcinoma Target Interactional Network Analysis

**FIGURE 3:**
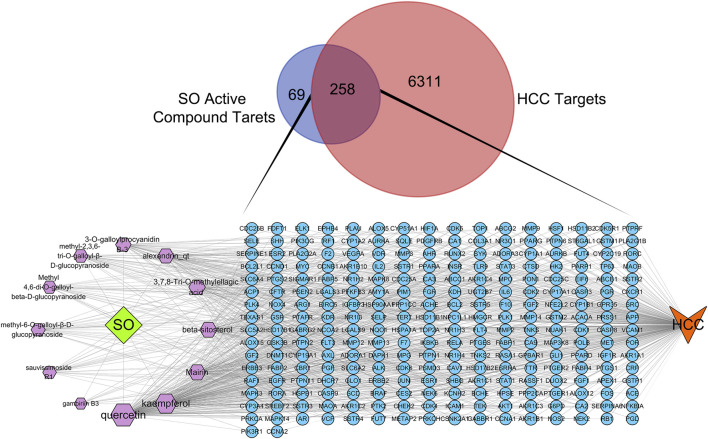
The Venn diagram of HCC targets and SO active compound targets and C-D-T network of SO. The blue circle stands for gene, purple hexagon stands for compounds of SO. The green diamond stands for SO, and orange V shape stands for HCC.

**FIGURE 4:**
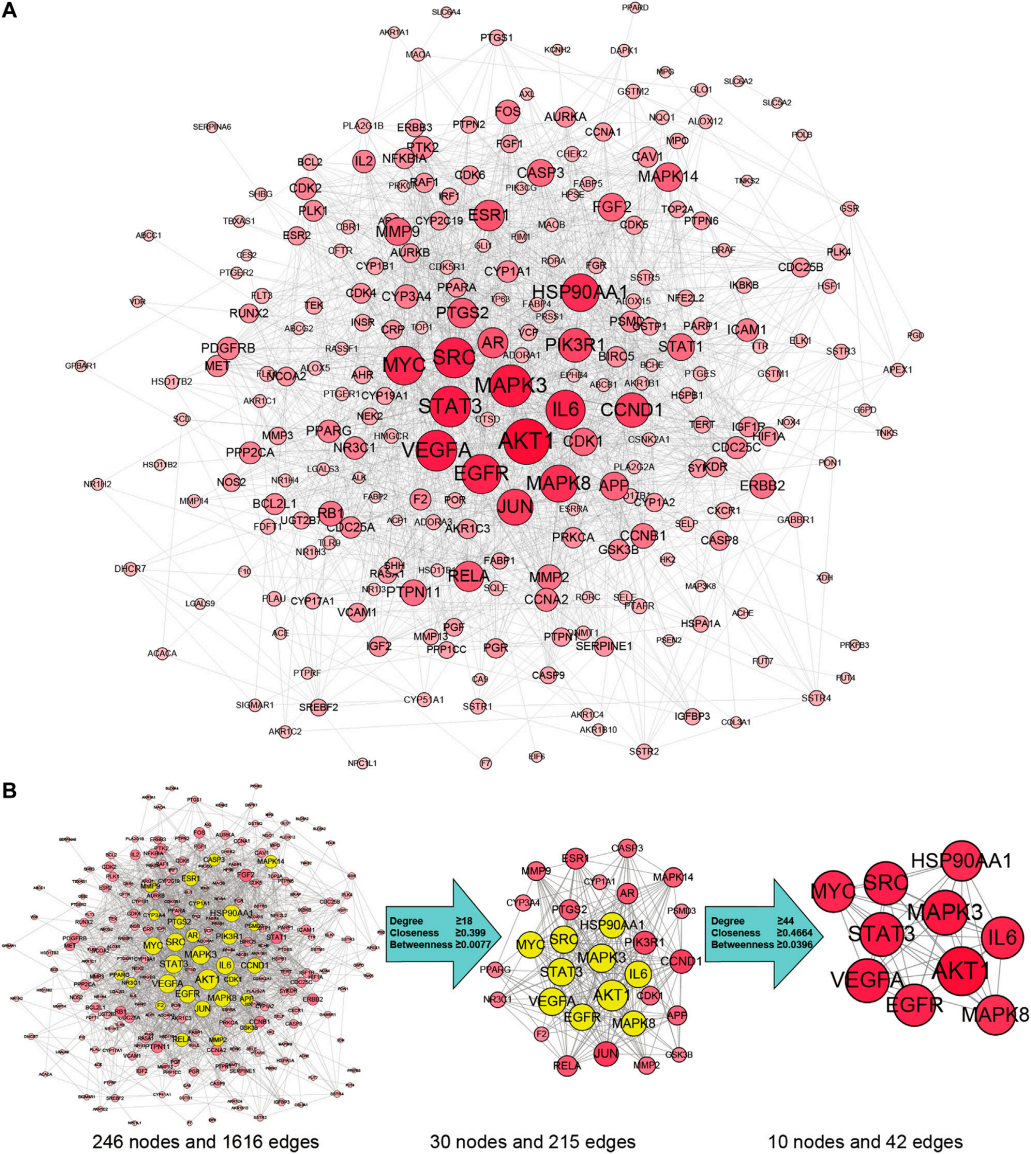
SO targets—HCC PPI network. **(A)** The network contains 246 gene nodes and 1,616 edges. Pink circle represents gene targets. The red nodes have higher degrees. Node size of gene targets is proportional to the number of degrees. **(B)** The process of hub network topological screening.

By searching the GeneCards, OMIM, TTD, PharmGKB databases, we obtained 6,569 genes associated with HCC. A total of 258 overlapping genes were obtained by looking for the intersection of the above compound target and the HCC targets (Figure 3. and Supplementary Table S2). By inputting compound–disease co-targets data into STRING, we obtained compound target–HCC target PPI network with higher connectivity (interaction score ≥0.700), containing 246 nodes and 1,616 edges (Figure 4A). The topological analysis of the PPI network, based on three major network parameters of “degree”, “betweenness” and “closeness”, was applied to select nodes above two-fold median values as key targets and constructed the hub nodes of the anti-cancer effect of SO on HCC. The threshold values of the first screening were degree≧18, closeness≧0.399 and betweenness ≧ 0.0077, and the results were 30 hub nodes and 215 edges. The 30 key targets were then further screened with the second screening threshold values as degree ≧ 44, closeness ≧ 0.4664, and betweenness ≧ 0.0396. The second screening ended up with 10 hub nodes and 42 edges (Figure 4B). In the hub network, the nodes interacted with others numerous edges (66 in AKT1, 59 in MAPK3, 57 in STAT3, 57 in VEGFA, 56 in SRC, 54 in IL6, 54 in EGFR, 53 in MYC, 51 in HSP90AA1 and MAPK8). These results suggested that these high-degree hub targets may account for the essential therapeutic effects of SO in HCC.

### GO Biological Process and KEGG Pathway Enrichment Analysis

**FIGURE 5:**
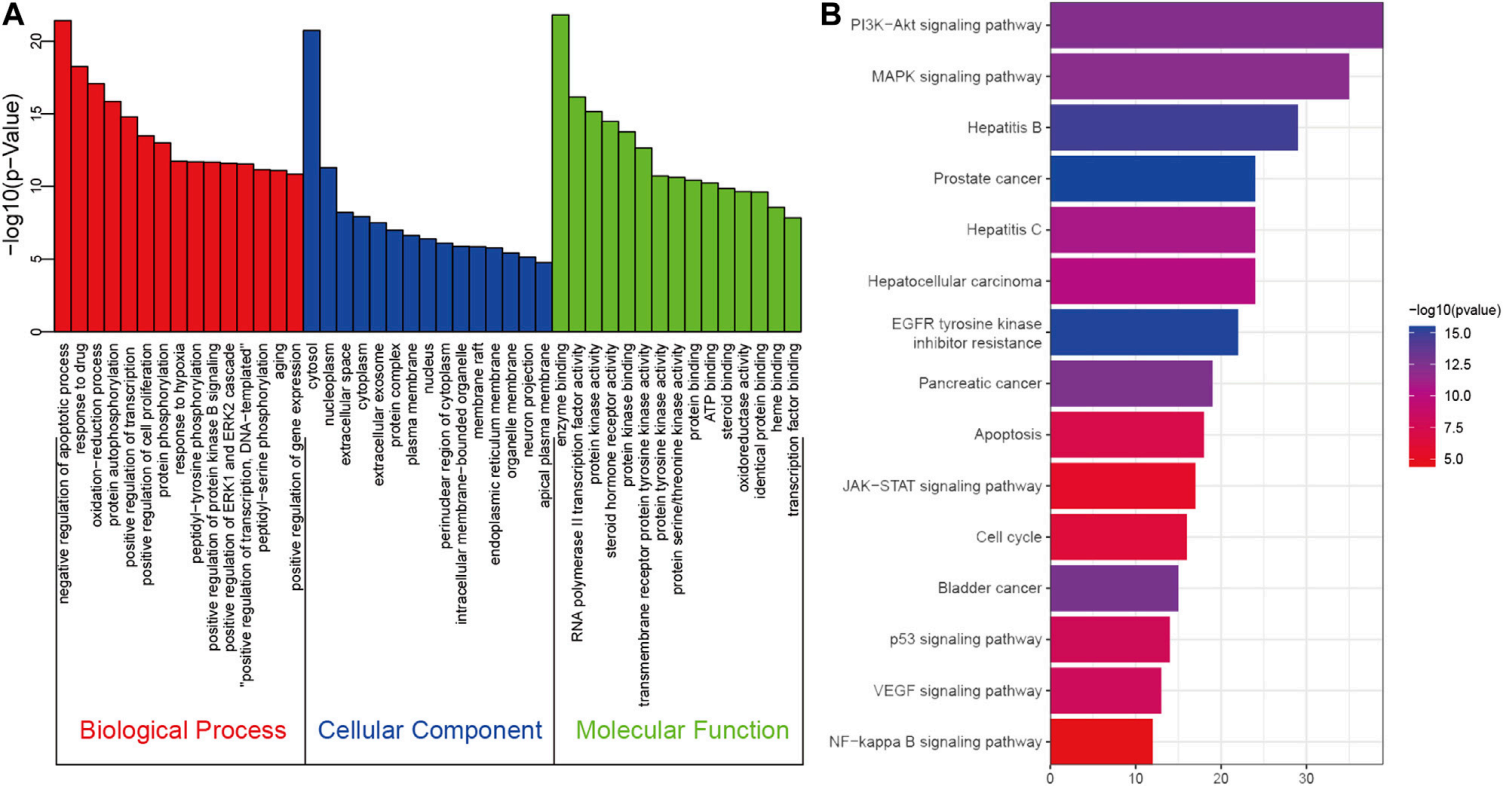
The GO **(A)** and KEGG pathway enrichment **(B)** analysis of 258 therapeutic genes of SO on HCC.

To explore the therapeutic mechanisms of putative targets of SO on HCC, the GO and KEGG pathway enrichment analyses were performed using DAVID 6.8. There were respectively 474 biological process (BP), 53 cellular component (CC), and 136 molecular function (MF) terms in total, which met the requirements of Gene count ≥2 and *p*-value ≤ 0.05 (Supplementary Table S3). The top 15 significantly enriched GO terms in BP, CC, and MF were plotted in Figure 5A, showing that SO may regulate HCC cancer cell proliferation and apoptosis via protein kinase binding, enzyme binding, and transcription factor binding in cytosol, nucleoplasm, extracellular, protein complex and plasma membrane to exert its anti-cancer effect on HCC. To further reveal the underlying mechanism on involved pathways of SO on HCC, KEGG pathway enrichment analysis of involved targets was conducted (Supplementary Table S4). The most significantly enriched 15 pathways of SO on HCC were shown in Figure 5B. The result also indicated the PI3K/AKT signaling pathways is the top enriched pathway, with 39 involved targets.

### Target-Pathway Network Analysis

**FIGURE 6:**
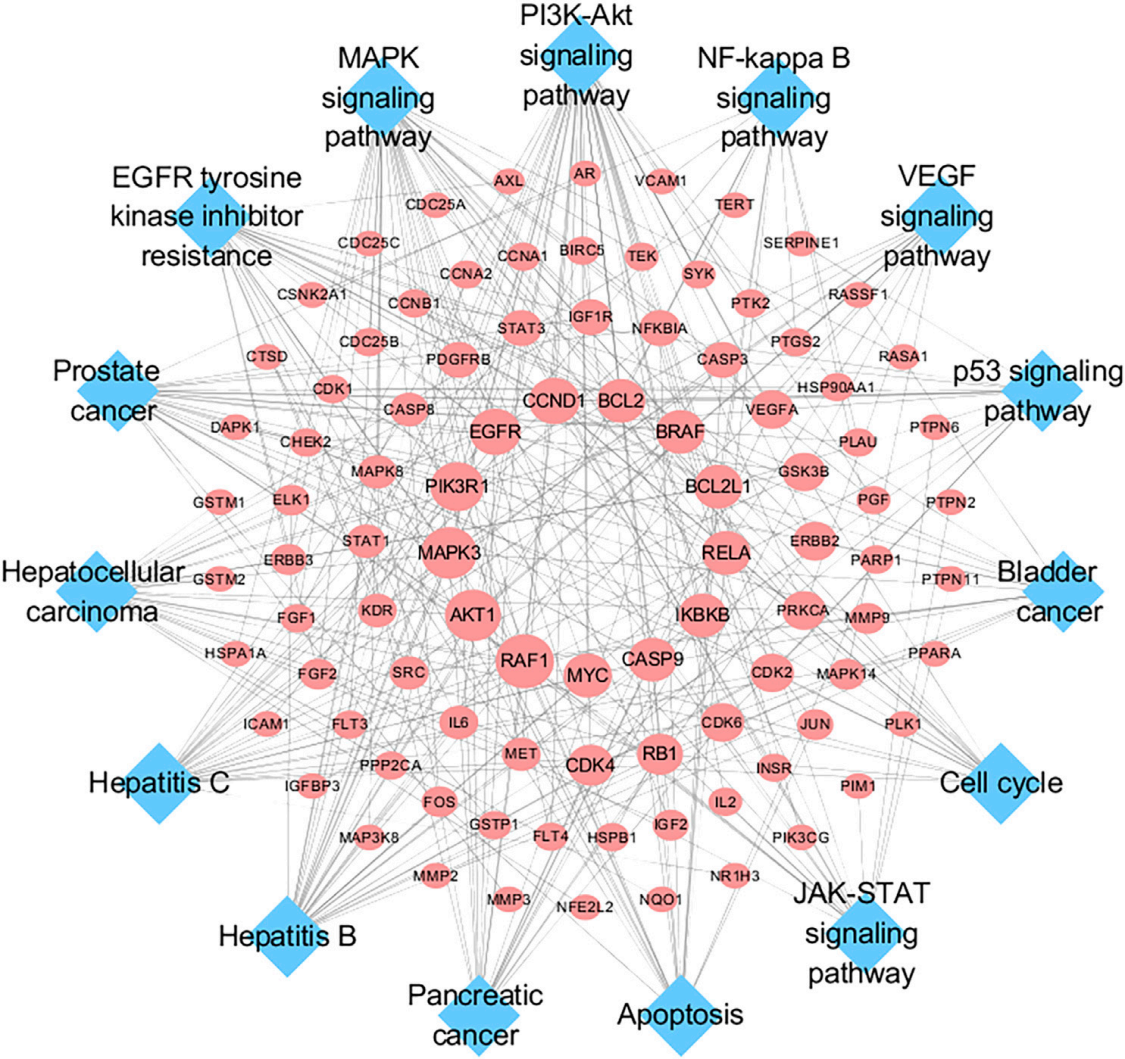
Target-Pathway Network. The red ellipse nodes represent the target nodes. The blue diamond nodes represent the corresponding pathways.

To further investigate the molecular mechanism of SO alleviated HCC, a target-pathway network was constructed based on top 15 significant signaling pathways and their corresponding genes (Figure 6). This network consisted of 109 nodes (94 genes and 15 pathways). In these pathways, PI3K/AKT signaling pathways is the top important one with the highest degree. Among these target genes, AKT1, PIK3R1, EGFR, MAPK3, CCND1, IKBKB, RELA, BRAF, BCL2, BCL2L1, CASP9, MYC and RAF1 were identified as relatively high-degree targets. Therefore, these genes were considered as the key therapeutic genes of SO on HCC. From the drug target prediction, GO and pathway enrichment analysis and pharmacology networks analyses, we suggested that the anti-cancer effects of SO on HCC might be related to regulate cancer cell proliferation and survival via pathways including EGFR, PI3K/AKT, NFκB and MAPK signaling pathways.

### *Sanguisorba Officinalis* L. Inhibits Hepatocellular Carcinoma Cell Proliferation and Induces Cellular Apoptosis

**FIGURE 7:**
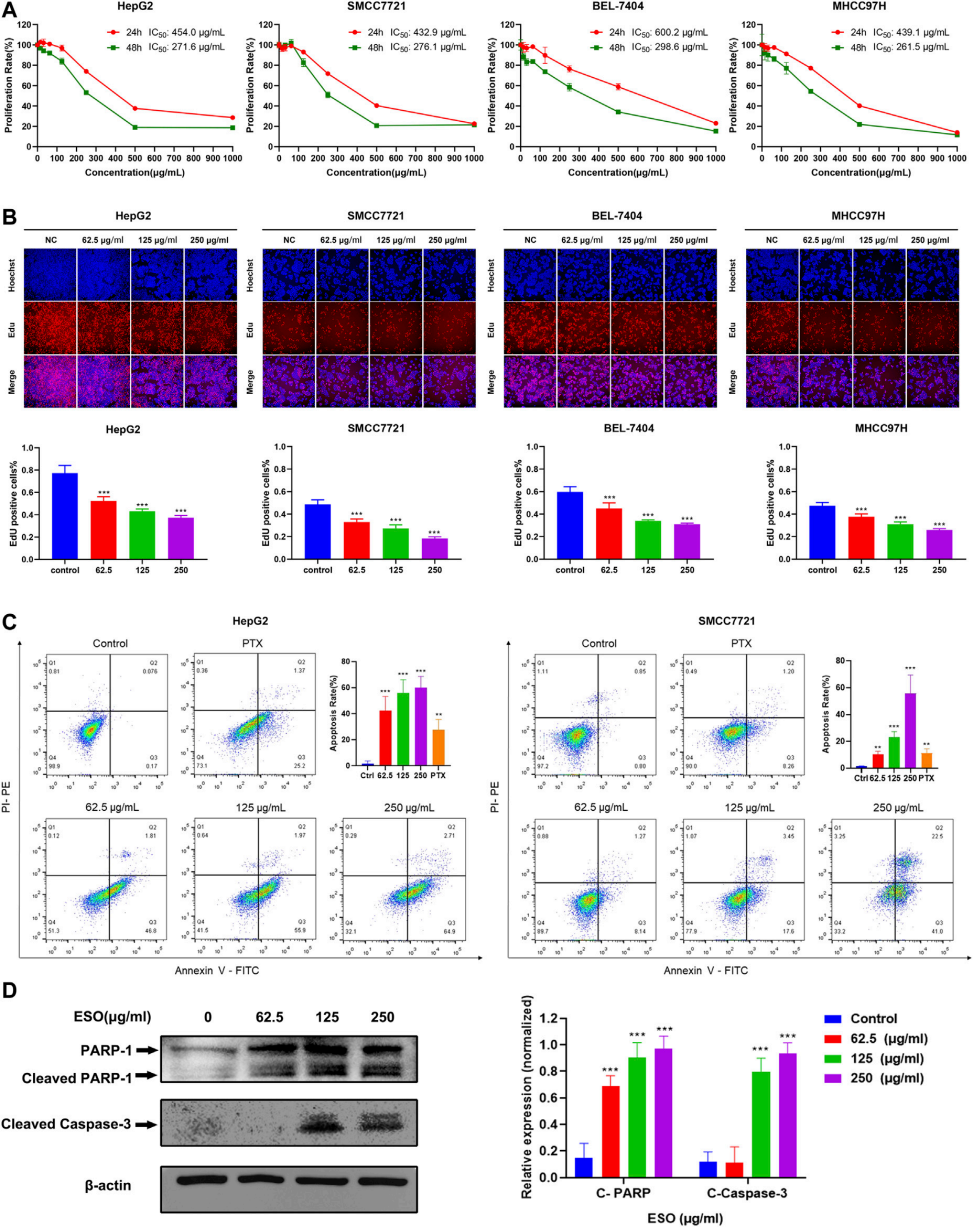
SO inhibits HCC cell proliferation and induces cellular apoptosis. **(A)** the cell viability was tested by CCK8 assay, showing a time- and dose-dependent inhibiting effect of ESO treatment on the viability of HCC cells. *n* = 3. **(B)** EdU assays were performed to verify the effects of ESO treatment on HCC cells proliferation, the rate of EdU-posive cells was calculated. *n* = 3. **(C)** Apoptosis analysis of ESO-treated Hepg2 and SMCC7721 cells was analyzed by flow cytometry after Annexin V-FITC/PI staining. *n* = 3. The concentration of PTX of positive control group was 40 nM, **(D)** The protein levels of PARA and cleaved Capase-3 in HepG2 cell. *β*-actin was used as an internal control. *p < 0.05, **p < 0.01, ***p < 0.001 vs. the nontreated control group.

To assess the anti-proliferative effect of SO on HCC as predicted from network pharmacology analysis, CCK8, Edu, and apoptosis flow cytometry were performed on several HCC cell lines. As shown in Figure 7A, ESO remarkably inhibited the viability of HCC cells (HepG2, MHCC97H, SMCC7721, and BEL-7404), in a dose- and time-dependent manner upon treatment for 24 h and 48 h. And according to the IC50 values, BEL-7404 is the least sensitive cell line for ESO, with other HCC cell lines showing almost same level sensitivities. As shown in Figure 7B, the EdU assay further confirmed that ESO reduced the percentage of proliferation active cells in a dose-dependent manner, after treatment with increasing ESO for 24 h. Figure 7C,D shows that the percentage of apoptotic cells and the apoptosis biomarker (cleaved PARP and cleaved capase 3) were notably increased in a dose-dependent manner, after treatment with ESO for 24 h. In summary, these findings clearly confirmed that ESO inhibit HCC cell proliferation in various aspects.

### *Sanguisorba Officinalis* L. Inhibits Human Hepatocellular Carcinoma Cell Migration and Invasion

**FIGURE 8:**
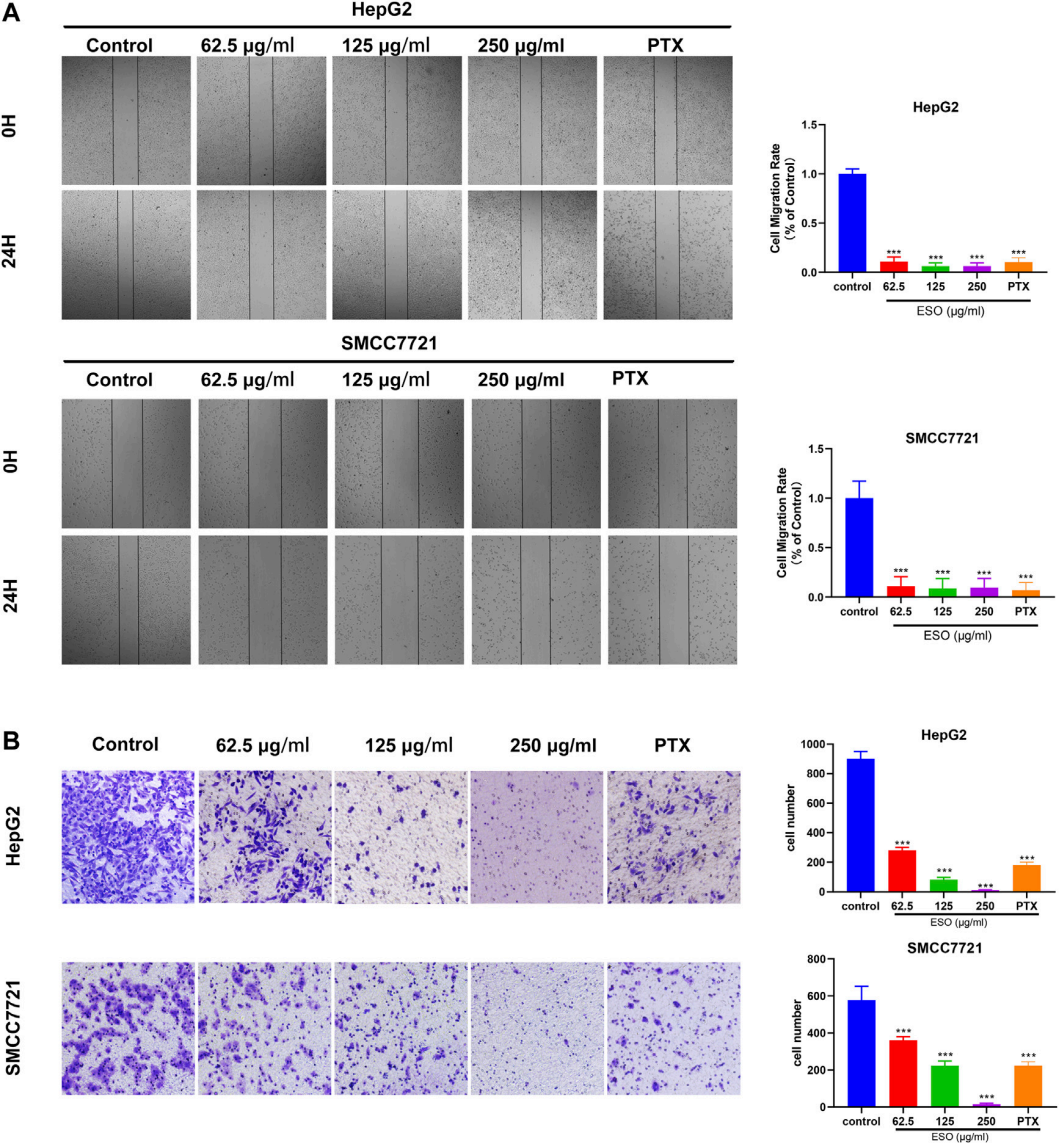
SO inhibits human HCC cell migration and invasion **(A)** Wound healing migration assay of HepG2 and SMCC7721 cells in the presence of ESO. *n* = 3. **(B)** Transwell invasion assay Hepg2 and SMCC7721 cells in the presence of ESO. *n* = 3. The concentration of PTX of positive control group was 40 nM, *p < 0.05, **p < 0.01, ***p < 0.001 vs. the nontreated control group.

To explore SO’s effect on migratory and invasive ability in human HCC cells, wound healing and transwell invasion assays were performed. As is shown in Figure 8A, compared to the control group, the migration of HepG2 and SMCC7721 cells was markedly inhibited in the ESO treatment group. As is shown in Figure 8B, in the transwell invasion assay, the invasion of ESO treated groups were inhibited in a dose-dependent manner. These results confirmed that SO suppressed the migration and invasion of HCC cells.

### *Sanguisorba Officinalis* L. Attenuated Proliferation Pathways in Hepatocellular Carcinoma Cells

**FIGURE 9:**
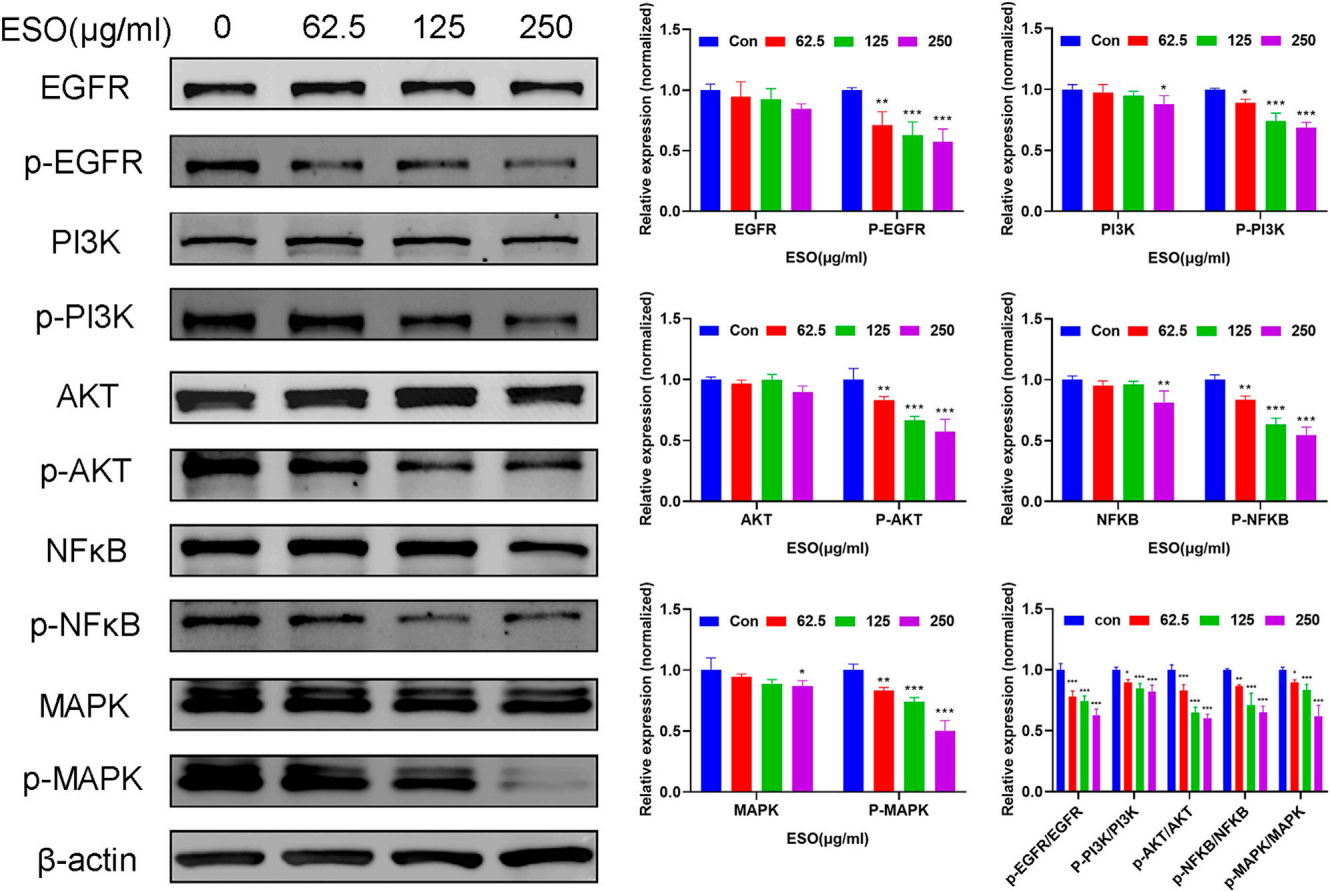
Western blot analysis of related proteins with ESO treatment in HepG2 cells *p < 0.05, **p < 0.01, ***p < 0.001 vs. the control group. Statistical results of protein levels ware normalized by control group.

Network pharmacology analysis described above suggested that the PI3K/AKT, EGFR, MAPK, and NFκB signaling pathway may be highly related to the anti-cancer mechanism of SO on HCC in regulating HCC cell proliferation and survival. Therefore, we further assessed the expressions level of the EGFR, PI3K, AKT, MAPK and their phosphorylated counterparts by western blot. As shown in Figure 9, pretreatment of HepG2 cells with ESO (62.5, 125 and 250 μg/ml) led to apparent repression of phosphorylation level of EGFR, PI3K, AKT, MAPK (p44, p42) and NFκB (p65) in a dose-dependent manner. Taken together, these results suggested that the effect of SO on HCC might be exerted via the EGFR/MAPK and EGFR/PI3K/AKT/NFκB pathways.

## Discussion

Development of HCC is a very complicated and multistep biological process, which is associated with diverse molecular and cellular signaling pathways (Forner et al., 2018). Owing to the complexity of compounds, TCM may show extensive pharmacological activities with multiple protein targets. However, because of this property, it is difficult to investigate the underlying mechanisms of TCM (Zhang et al., 2019). Network pharmacology, derived from systems biology and bioinformatics, provide a comprehensive and powerful approach for studying the mechanism of complicated TCM (Zhou et al., 2020). In the current study, we applied network pharmacology approach to predict the pharmacological mechanism of SO on HCC, following validation by experimental methods.

Pharmacokinetic properties are very important for drug screening and design. Due to lacking suitable pharmacokinetic properties, drugs could not reach the target organs to exert the therapeutic effect (Tian et al., 2015). In this study, 12 chemical components of SO were identified by the criteria of oral bioavailability ≥30% and drug-likeness index ≥0.18, because of their good absorption and druglike feature. Among these 12 active components, several compounds have been reported to possess anti-cancer effects including HCC. Among of them, quercetin, a natural flavonoid, was reported to exert an anti-carcinogenic effect via increasing p53 and BAX and downregulating ROS, PI3K, COX2 and PKC in HCC cell line (Maurya and Vinayak, 2015). And it was also reported that quercetin exert its anti-cancer effects by promoting apoptosis and autophagy through the modulation of PI3K/Akt/mTOR, Wnt/-catenin, and MAPK/ERK1/2 pathways (Reyes-Farias and Carrasco-Pozo, 2019). Kaempferol, a phytoestrogen, was reported to induce autophagic cell death and inhibit survival and proliferation of human HCC cell lines through targeting AMPK signaling pathway (Han et al., 2017). Moreover, kaempferol treatment was shown to significantly inhibit HIF-1 activity and HCC cell viability under hypoxic conditions (Mylonis et al., 2010). And mairin, also called betulinic acid, a lupane-type pentacyclic triterpene, was shown to induce apoptosis and suppressed metastasis in both HCC cells and NOD/SCID mice model (Wang et al., 2019). All these literatures together with our experimental studies supported the conclusion of network pharmacology prediction and shown a good practice of network pharmacology method in identification of function mechanism of TCM herb.

From the integrated network pharmacology analysis, SO might exert anti-HCC effects by suppressing of cancer cell proliferation and survival, which was reported as the crucial mechanism of HCC development (Llovet et al., 2015). As demonstrated by network pharmacology analysis, SO may exert anti-HCC effects mainly by inhibiting proliferation and survival on HCC via EGFR, PI3K/AKT, NFκB, MAPK signaling pathways. To further verify the prediction, various *in vitro* experiments were performed to investigated the therapeutic effects of SO on diverse HCC cells, showing that ESO treatment significantly suppressed HCC cell proliferation, induced cellular apoptosis, and inhibited the cell migration and invasion activities in a dose-dependent manner. Especially, the phosphorylation level of EGFR, PI3K, AKT, MAPK and NFκB (p65) were significantly downregulated in a dose-dependent manner. EGFR, a member of the epidermal growth factor receptor family on cell surface, binds epidermal growth factor to trigger tyrosine kinase phosphorylation, leading to cell proliferation. It was reported that EGFR signaling axis exert a major regulatory effect during liver regeneration, liver cirrhosis and HCC, showing a pivot role of EGFR signaling in the development of liver diseases (Komposch and Sibilia, 2015). Thus, after HCC cells were treated by ESO, the downregulated phosphorylation of EGFR may contribute to its anti-cancer effects. PI3K is a kinase which could phosphorylate the inositol ring of phosphatidylinositol and related second messengers, coordinating a variety of cell functions including proliferation and survival. It was reported that the PI3K can be activated by many oncogenes and growth signaling, and upregulated PI3K signaling is regarded as a hallmark of cancer (Fruman et al., 2017). AKT, the major downstream target of PI3K, is a key pivot kinase in signaling communication network and it makes crosstalk between several important pathways, including NF-κB, p53, apoptosis and cell cycle pathways, regulating diverse important cellular processes including cell growth, survival regulation and metabolism in multiple solid tumors (Whittaker et al., 2010). Moreover, it was reported that PI3K/AKT/mTOR signaling pathway is strongly related to HCC development and the inhibition of PI3K signaling axis could be a HCC treatment strategy (Dimri and Satyanarayana, 2020). From our validation in laboratory, after pretreatment with ESO, the apparent decrease of pPI3K and pAKT in HCC cells might contribute to its anti-cancer effects. NFκB, a pleiotropic transcription factor, plays an essential role in inflammation, cell growth, immunity, differentiation, tumorigenesis and apoptosis. The abnormal activation of NFκB are highly associated with cancer development and progression, and the signaling pathways that induce NFκB activation provide promising targets for chemotherapeutic approaches (Karin and Greten, 2005; Karin, 2006). RELA, the 65KD subunit of NFκB, forms the most abundant heterodimeric NFκB complex with NFKB1. It was reported that antisense RELA oligomers exerted antigrowth effects on diverse cancer cells *in vitro* and caused a significant inhibition of tumorigenicity in nude mice tumor models (Sharma and Narayanan, 1996). In this research, target-pathway network shown that RELA, is one of the high-degree targets of SO. And in laboratory experiment, after pretreatment with ESO, a gradually decrease of pNFκB-P65 level in HCC cells was observed in a dose-dependent manner, which might contribute to anti-tumor effects of SO on HCC. Taking EGFR, PI3K, AKT, and NFκB together, Our finding was in accordance with the report that EGFR activation could further trigger the PI3K-AKT-NFκB signaling axis and eventually cause tumor cells proliferation (Engelman et al., 2006; Zhang et al., 2014). MAPK, also known as extracellular signal-regulated kinases (ERKs), act in a signaling cascade that regulates various cellular processes such as proliferation, angiogenesis,differentiation, apoptosis and survival in response to a variety of extracellular signals (Le Gallic et al., 1999). Moreover, it was reported that MAPK phosphorylation level in HCC tissues was 7 times higher than that in adjacent benign tissues (Huynh et al., 2003). And MAPK signaling activation might be caused by the upstream signals, such as EGFR signaling (Zhang et al., 2017). In our laboratory experiment, after treated with ESO, an apparent downregulation of pEGFR and pMAPK1 in HCC cells was observed, which may might also contribute to its anti-HCC effects.

**FIGURE 10:**
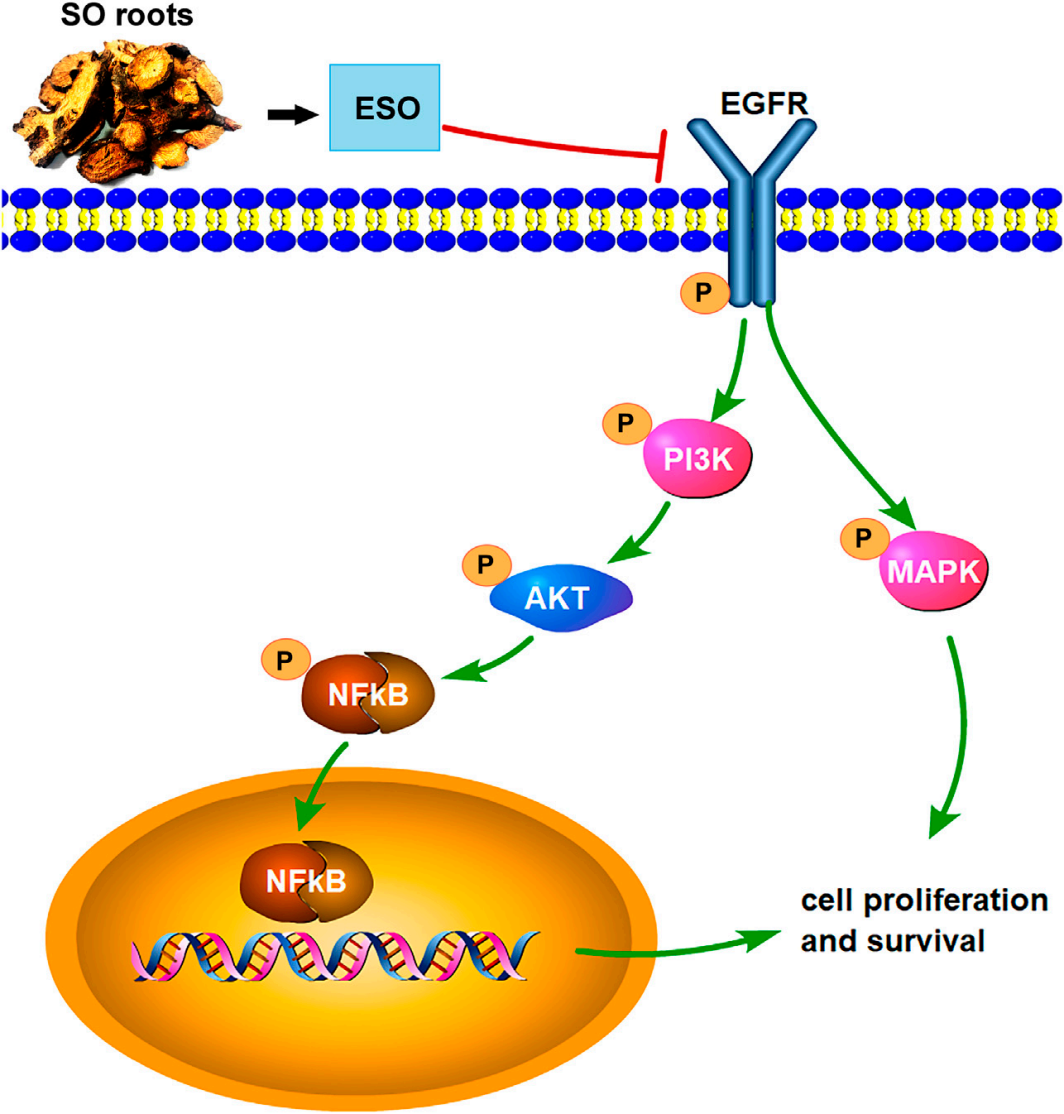
The overview of the regulatory mechanism involved in the anti-cancer effect of SO on HCC.

Taken together, this study for the first time, shows that SO can efficiently suppress cellular proliferation, migration, invasion and induce apoptosis in HCC cells, indicating that SO may inhibit HCC mainly through the EGFR/PI3K/AKT/NFκB and MAPK signaling pathways (Figure 10). Owing to budgetary and time constraints, the present study has some limitations. Firstly, the public online databases we used in this research are imperfect and constantly updating, so some of the active ingredients of SO and their target genes might not be included in the analysis. Moreover, there are other signaling pathways (e.g., VEGF, p53 and JAK-STAT signaling pathways) might also play roles in the anti-tumor effect of SO. Further studies are needed to explore the potential function of these pathways. Despite the limitations, this study provides powerful tool and preliminary data for further investigation of SO in HCC. SO might be a potential anti-HCC medicine, which can be developed as a therapeutic option for the treatment of HCC.

## Conclusion

In conclusion, via the integrating network pharmacology and experimental validation, our study has investigated the underlying mechanism of SO in suppressing HCC. The results suggest that SO might inhibit the proliferation, induce cellular apoptosis and impair the migration and invasion of HCC cells, mainly via regulating of EGFR/PI3K/AKT/NFκB, and MAPK signaling pathways. Moreover, the combined network pharmacology analysis and experimental validation in this study may provide a powerful tool to explore the mechanism of TCM.

## Data Availability

The raw data supporting the conclusion of this article will be made available by the authors, without undue reservation, to any qualified researcher.
