# Supplementary material for: Network Pharmacology and Pharmacological Evaluation Reveals the Mechanism of the Sanguisorba Officinalis in Suppressing Hepatocellular Carcinoma
Source: Front Pharmacol. 2021 Mar 4;12:618522. doi: 10.3389/fphar.2021.618522 (PMC7969657; doi:10.3389/fphar.2021.618522)
Supplement: Supplementary file 1 [file datasheet1.pdf]

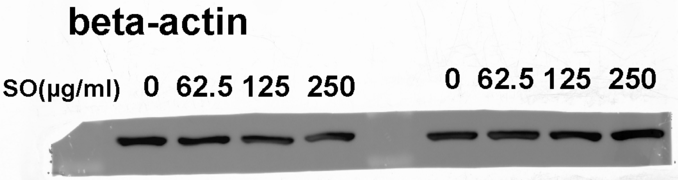
beta-actin
SO(μg/ml)
0
62.5
125
250
0
62.5
125
250

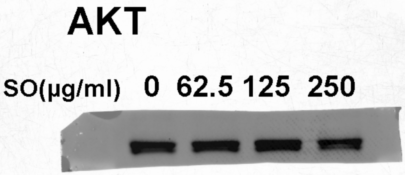
AKT
SO(µg/ml) 0 62.5 125 250

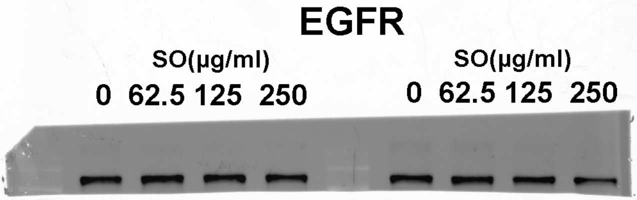
EGFR
SO(μg/ml)
0 62.5 125 250
SO(μg/ml)
0 62.5 125 250

SO(μg/ml) 0 62.5 125 250

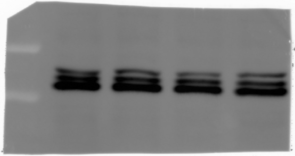

MAPK

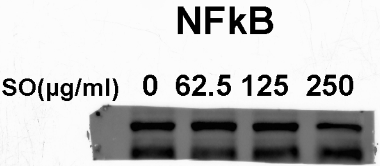
NFkB
SO(μg/ml) 0 62.5 125 250

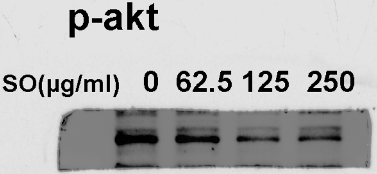
p-akt
SO(μg/ml) 0 62.5 125 250

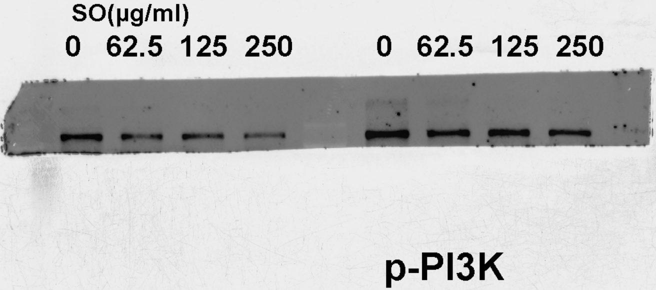

SO(μg/ml)
0 62.5 125 250 0 62.5 125 250
p-PI3K

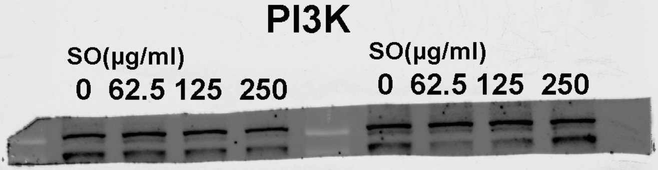
PI3K
SO(μg/ml)
0 62.5 125 250
SO(μg/ml)
0 62.5 125 250

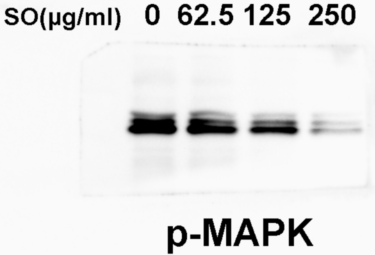
SO(μg/ml) 0 62.5 125 250
p-MAPK

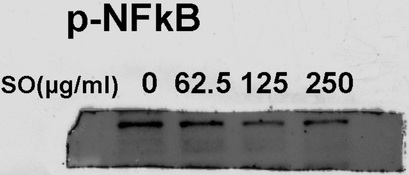
p-NFkB
SO(μg/ml) 0 62.5 125 250

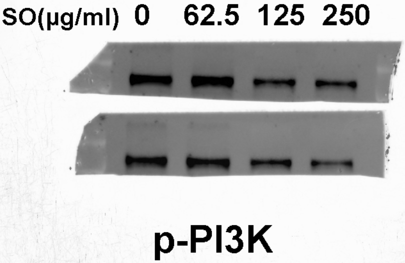
SO(μg/ml) 0 62.5 125 250
p-PI3K
