# Supplementary material for: Network Pharmacology and Pharmacological Evaluation Reveals the Mechanism of the Sanguisorba Officinalis in Suppressing Hepatocellular Carcinoma
Source: Front Pharmacol. 2021 Mar 4;12:618522. doi: 10.3389/fphar.2021.618522 (PMC7969657; doi:10.3389/fphar.2021.618522)
Supplement: Supplementary file 2 [file image1.pdf]

Supplementary Figures

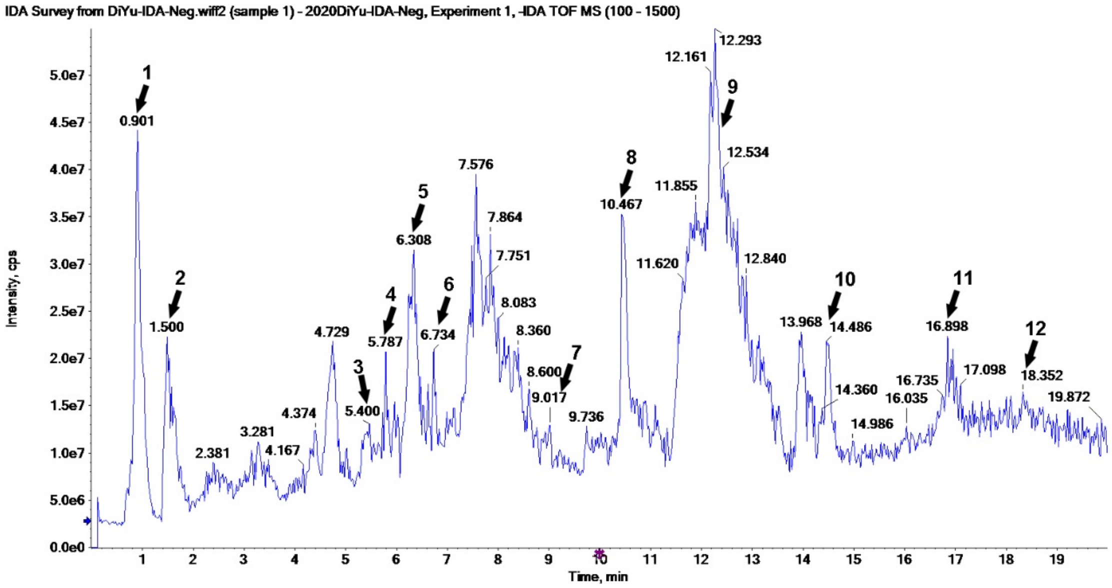

Supplementary figure 1. UPLC-Q-TOF/MS total ion chromatograms of ESO under negative ion mode. 1. 3,7,8-Tri-O-methylellagic acid; 2. Quercetin; 3. methyl-6-O -galloyl-β-D-glucopyranoside; 4, methyl-2,3,6-tri-O-galloyl-β-D-glucopyranoside; 5. kaempferol; 6. beta-sitosterol; 7. methyl 4,6-di-O-galloyl-beta-D-glucopyranoside; 8. gambiriin B-3; 9. 3-O-galloylprocyanidin B-3; 10. sauvissimoside R1; 11. alexandrin_qt; 12. mairin.
